# Performance differences with and without differential item functioning in the post graduate admission test in Saudi Arabia based on gender and ability level

**DOI:** 10.3389/fpsyg.2025.1515316

**Published:** 2025-10-20

**Authors:** Eqbal Z. Darandari, Muna A. Almeri

**Affiliations:** College of Education, King Saud University, Riyadh, Saudi Arabia

**Keywords:** differential item functioning (DIF), Mantel Haenszel method, gender gap, postgraduate general aptitude test (PGAT), High stake test, ability level

## Abstract

This study aimed to investigate Differential Item Functioning (DIF) based on gender and ability level for Post-Graduate General Aptitude Test (PGAT) items in Saudi Arabia, using classical methods (MH *χ*^2^, MH-LOR, BD *χ*^2^, and CDR). The study samples consisted of (4,000) students distributed equally between males and females. For overall sample, 56 (54%) out of 104 items showed DIF: with (48%) of them favoring females and (41%) favoring males. For high ability sample, percentage of DIF items decreased across subtests, particularly for verbal sub-test. DIF items favoring females decreased (to 40%) and the ones favoring males increased (to 55%). ANOVA results showed that for overall sample, females outperformed males on total score and verbal ability, while males outperformed females on quantitative and logical abilities, significantly (*p* < 0.01). When DIF items were removed for overall sample, gender gap was reduced except for verbal ability, favoring females. For high ability sample, differences on total and sub-scores were not statistically significant except for quantitative ability, that favored males (*p* < 0.01). When DIF items were removed for high ability sample, gender differences were not statistically significant (*p* > 0.05). Thus, it was recommended to conduct stratified DIF analysis for ability admission tests based on ability area and level, gender and their interaction; and to report DIF size and direction for ability groups based on cut scores.

## 1 Introduction

Differential Item Functioning (DIF) analysis is part of test construction and validity. It ensures that the scores of individuals on the test reflect the same structure or composition for individuals with equal abilities on measured traits (Walker, 2011). The absence of item differential performance is one of the most important conditions that must be met in the test before its publication. Conducting DIF for items of large-scale tests, and understanding their sources became a routine part of test development (Gierl et al., 2001). It is required by a number of educational associations interested in test development, within their publication rules for tests used in decision-making, and they indicated the need for clear empirical evidence to support the absence of DIF items across important groups, because the presence of DIF items can affect the validity and comparability of the scores for intended uses and interpretations. The American Educational Research Association (AERA), the American Psychological Association (APA), and the National Council for Measurement in Education (NCME), have considered DIF a necessary standard when preparing and publishing tests (AERA, APA and NCME, 2014).

DIF occurs when the performance of test takers from a certain group (for example, gender, region, ethnicity) differs significantly on certain items compared to the reference group (Setiawan et al., 2024). Subgroups that are typically included in DIF studies are: gender, school type, religion and socioeconomic status (Liu and Bradley, 2021).

### 1.1 Differences in the performance on cognitive ability tests based on gender and ability

There are a number of studies that have revealed statistically significant gender differences, and many of them have shown that women outperformed men on verbal abilities, while men outperformed women on quantitative and logical abilities, across age groups and countries, with considerable magnitude (Hyde and Linn, 1988; Hyde and Mertz, 2009). However, most of these studies did not fully consider methodological aspects and whether gender differences are real differences or due to DIF. On the other hand, some studies examined gender differences and DIF in cognitive ability achievement tests using different DIF methods as Setiawan et al. (2024) reported; indicating, in general, that DIF favored males on mathematical skills and females on verbal ability skills (e.g., Abedalaziz, 2010; Al-Bursan, 2013; Al-Bustanji, 2004; Almaskari et al., 2021; Kalaycioglu and Berberoglu, 2011; Yörü and Atar, 2019; Wedman, 2018).

Other studies indicated that DIF direction and percentage may differ in cognitive tests based on ability level. For example, in Shanmugam’s (2020) study the results showed that computation items with one step operation, which assess lower-order thinking skills favored females, while items that assess higher-order thinking skills favored males. In addition, a study conducted by Al-Bursan (2013) reported that the percentage of DIF items increased as student ability decreased, and gender differences in mathematics were most prominent at the very high levels of ability and were content and ability dependent.

### 1.2 DIF in ability admission tests based on gender and ability

Gender differences in the performance on high-stakes assessments, of high impact, have more attention from researchers to evaluate whether a test is fair or not. However, tests that are used for admission have received less attention than general intelligence and ability tests in DIF studies; where limited studies investigated DIF in the admission and other academic selection tests, such as the Scholastic Assessment Test (SAT) and the Graduate Record Exam (GRE) (Scheuneman and Gerritz, 1990). Given the important practical implications of the results of college admission tests, it is important to investigate their fairness for all targeted groups. New studies strongly advised to have protocol for admission tests to enhance equity and diversity in higher education (Woo et al., 2023). Shamsaldeen et al. (2024) indicated that college entrance admission exams became controversial, regarding the fairness of test scores. Poorly formatted items, improper item content, or measuring an irrelevant construct are from the reasons that may cause DIF in them.

The few studies (e.g., Alavi and Bordbar, 2017; Freedle and Kostin, 1990; Gu et al., 2006; Kalaycioglu and Berberoglu, 2011; Pae, 2004a, 2004b; Setiawan et al., 2024; Shamsaldeen et al., 2024; Sideridis and Tsaousis, 2013a, 2013b; Tasaousis et al., 2023) used diverse detection methods and grouping variables to investigate DIF of admission tests in colleges and universities around the world. For example, Sideridis and Tsaousis (2013a, 2013b) conducted studies to examine DIF by gender (males and females), school type, region, of the General Aptitude Test (GAT) items for high school students, provided by the National Center for Assessment (NCA) in Saudi Arabia. They conducted the first study on science specializations, and the second on literal arts specializations, using the area between the item characteristics curves method, with 1-parameter. The results for science specializations showed non-uniform DIF in 2 items, according to gender in favor of females with low ability level, and 2 items in favor of government school students with low ability. As for literal arts specializations, the results showed non-uniform DIF in 2 items based on gender in language, non-uniform DIF in one item according to the school type, and a uniform DIF in one item in favor of low-ability government school students.

Besides, Tsaousis et al. (2020) examined DIF in Chemistry sub-test of the Standard Achievement Admission Test (SAAT) in Saudi Arabia universities, using odds ratio method. The sample consisted of (6,265) students, and they found that DIF existed for five items, three items (number 45, 54, and 61), were moderate, and two items (number 51and 60) were strong. Tasaousis et al. (2023) also conducted a study for DIF based on gender on SAAT for the Science. They used Multiple Indicators Multiple Causes (MIMIC) approach with General Aptitude Test (GAT) to explain DIF. The sample consisted of (1,300) Saudi high school students. The results showed that (13) items exhibited DIF effects for different gender groups, favoring males, and GAT helped in explaining the DIF.

In Kuwait, Shamsaldeen et al. (2024) investigated DIF in the Kuwait University English Aptitude Test (KUEAT). The sample consisted of (1,790) examinees, and the results revealed many items showing DIF across student sub-population groups (i.e., nationality, gender, high school majors, and high school types). Moreover, Setiawan et al. (2024) investigated DIF in the National Examination Questions in mathematics for high schools in the Yogyakarta region, as a reference group, and the South Kalimantan region as a focus group sample for a sample of (1,000) student for each; using the Likelihood Ratio Test (LRT) method, Area Measure Raju, and Lord. The results showed that by using the LRT method, the researchers found 36 items had significant DIF detection, and 32 items were significant by Raju Area method.

For the post-graduate demission tests, less DIF studies were conducted based on gender. For example, studies showed a history of gap differences on GRE (that measures verbal reasoning, quantitative reasoning, and analytical writing abilities) based on gender (Hirschfeld et al., 1995). In Saudi Arabia, a similar admission test to GRE is administered for the same purpose with similar sections called Post-Graduate General Aptitude Test (PGAT). The few studies (such as: Sideridis and Tsaousis, 2013a, 2013b; Tsaousis et al., 2020; Tasaousis et al., 2023) that were conducted on DIF for cognitive ability and achievement admission tests, were for high school students as predictors of their academic achievement.

Despite the importance of PGAT for students as a criterion for post-graduate admission for Saudi universities, the psychometric properties published about the test are still insufficient, and none of the studies dealt with gender and ability differences and DIF in PGAT. Given the importance of PGAT in measuring ability and making decisions related to students, checking the presence of DIF in respect to gender (male/female) is essential. Thus, the present study aimed to investigate whether gender differences on PGAT can be attributed to actual gender differences or to DIF, and whether they differ across ability levels.

### 1.3 DIF methods and types

DIF is a statistical characteristic of an item that shows the extent to which the item might be measuring different abilities for members of separate subgroups. Hambleton and Rogers (1989) defined item DIF as the differences in the probabilities of the correct answer to the item for different groups of equal ability.

The concept of DIF is not synonymous with the concept of bias. It is considered an essential but insufficient condition to regard an item as biased, since DIF is a metric characteristic of the item; whereas bias refers to the theoretical explanation for its presence (Camilli and Shepard, 1994; Clauser and Mazor, 1998). Thus, DIF could be considered as an indicator of bias observed when test takers from different groups have different probability or likelihood of responding correctly to an item, after controlling for ability. For example, in the two-group case, the group that is concerned about items being biased against is called the focal or target group, and the other group, is the reference group. The focal group is the focus of the analysis, and the reference group serves as a basis (Liu and Bradley, 2021).

Several methods were developed to detect DIF, such as Mantel Haenszel Method (MH), Transformed Item Difficulty Method (TID), Analysis of Variance (ANOVA) Method, Logistic Regression Method, Item Discrimination Method (IDM), Chi-Square Method, Distracter Response Analysis, Item Characteristic Curve (ICC), b—Parameter Difference Method, and Likelihood Ratio Method (Ajmi et al., 2023). Newer methods include comparing more than two groups, such as the Generalized Logistic Regression, or grouping items, such as bundle items methods (Ibrahim, 2024). The size of DIF could be classified into small, medium, and large, using different measures of effect size depending on the method used to detect it (Ajmi et al., 2023).

The DIF methods could be classified based on psychometric theories: Classical Test Theory (CTT) and Item Response Theory (IRT). They could also be classified into parametric and nonparametric methods (Li and Becker, 2021; Ohiri et al., 2024; Lim et al., 2021). When choosing among DIF methods, there are some considerations: (i) how items were scored; (ii) type(s) of DIF to be detected; and (iii) sample size (Ibrahim, 2024).

DIF could also be divided into two types: uniform and non-uniform DIF. Uniform DIF occurs when the difference in item response between the two groups (reference and target) favors one group constantly across all levels of ability. Non-uniform DIF occurs when the difference in item response between the two groups of individuals is not consistent and varies across all levels of ability; or the probability of correctly answering an item is higher for one group at some points on the scale, and higher for the other group at other points (Walker, 2011).

### 1.4 DIF Mantel Haenszel (MH) method and procedures

One of the famous CTT methods that is widely used in investigating DIF is Mantel Haenszel (MH) method, particularly in educational testing based on gender (Ajmi et al., 2023; Navarro-González et al., 2024). Mollazehi and Abdel-Salam (2024) stated that MH is widely utilized for its simplicity, practicality and effectiveness in detecting uniform DIF.

MH method was presented by Mantel and Haenszel (1959), and was applied by Holland and Thayer (1988). It provides both the statistical significance and an estimate of the effect of the differential performance of the item (Holland and Thayer, 1988). Despite the fact that this method is classical, it is widely used because it does not require specific forms of item response functions. In addition, it is easy to compute and implement, and has the capacity of handling small sample sizes, particularly when percentage of DIF items in a test is not high, DIF magnitudes are large, and when there is no mean ability difference between groups (Jin et al., 2018; Narayanon and Swaminathan, 1996; Ukanda et al., 2019). MH χ^2^ tests the null hypothesis that there is no relationship between the individuals’ membership in a group and their performance on the test. It depends on the difference in the percentage of correct and wrong answers, between the target and reference groups, at each level of ability, using the total score of the test. One of the groups is called the reference group and the other is called the target group, which is the group that is believed to be affected by the differential performance of the item.

Several meta-analysis studies investigating the effectiveness of MH method when used to detect DIF (Guilera et al., 2013; Maranon et al., 1997) showed that MH procedure displayed adequate statistical power and type I error rates, especially when the sample size was (500) and above. It was more effective when purification procedures were used, and test contamination was below 20%. Moreover, when the size of DIF increased, discarding items with DIF from the test increased the estimation error, and when the ratio of items with DIF increased, the ability estimations differed in individual and group levels (Camilli and Shepard, 1994).

The agreement between MH approach and other DIF approaches was investigated. Ajmi et al. (2023) examined DIF of verbal ability test items in Multiple Mental Abilities Scale by gender (male vs. female) and country (Oman vs. the rest of the Gulf countries) using the MH and the LRT. The sample consisted of (2,688) students in grades five and six. The results showed that (16.7%) of items had DIF in relation to gender, and (33.3%) based on country. The agreement between the MH approach and LRT for both gender and country was quite high (73 and 66%, respectively).

In addition, other decision making statistical tests are used with MH. Breslow-Day (BD) χ^2^ (Breslow and Day, 1980; Penfield, 2003) is usually used in identifying non-uniform DIF (depending on ability or group membership) as it assesses odds ratio heterogeneity trends; and has proved to be effective (Prieto-Marañón et al., 2012). Besides, Log-Odds Ratio (MH-LOR) is used to indicate whether an item favors reference group or target group. It is asymptotically normally distributed, where positive values indicate that DIF is in favor of the reference group, and negative values indicate that DIF is in favor of the target groups. In addition, the Combined Decision Rule (CDR) that combines MH with the BD is used to flag any item for which either the MH *χ*^2^ or BD *χ*^2^ is significant in order to increase the power to detect DIF (Penfield, 2013). Penfield (2003) conducted a simulation study to evaluate the statistical power and the rate of type I error of BD and CDR, and the results showed that the CDR performance was better under various conditions regarding the type I error rate and the statistical power compared to others. Mollazehi and Abdel-Salam (2024) also showed that the adjusted MH detection rates were comparable to those of other standardized procedures like BD and even better.

Many studies used MH method only to detect uniform DIF, neglecting the non-uniform type (e.g., Al-Bursan, 2013; Al-Etawi, 2004; Innabi and Dodeen, 2006); while a few studies combined MH and other methods (such as BD) to detect uniform and non-uniform DIF to overcome the inflation of type I error (e.g., Abedalaziz, 2010; Al-Bustanji, 2004; Chiu, 2008; Hambleton and Rogers, 1989; Kalaycioglu and Berberoglu, 2011; Kelecioglu et al., 2014; Pedrajita and Talisayon, 2009; Penfield, 2003; Salubayba, 2013). Al-Bustanji (2004), Chiu (2008), and Penfield (2003) were among the studies that combined MH *χ*^2^ and BD *χ*^2^ methods to detect the uniform and non-uniform DIF, and showed that they were effective, particularly when targeting low rates of type I error.

Furthermore, Elyan and Al jodeh (2024) investigated the effectiveness of the MH-LOR method in detecting DIF based on gender, while considering variations in sample size and test length. The results showed that its ability to detect DIF items increased with larger sample sizes, while maintaining a consistent test length. However, there was a decline with longer test lengths, despite maintaining a fixed sample size at a specific level.

Thus, it is recommended that DIF studies for items use more than one method to combine the strengths and reduce the weaknesses of each method and to detect different types of DIF (Camilli and Shepard, 1994) and consider ability level. It is recommended also to use conditional DIF methods, particularly when overall ability differs among groups (Moses et al., 2010). In addition, some factors; such as sample size, could affect DIF. Alomari et al. (2023) investigated the effect of sample size on the number of items with DIF using the MH method, and indicated that more items were found using DIF as the sample size increased. More significant sample sizes are also required to detect non-uniform DIF. Sample sizes of 200 to 250 per group will likely have enough power to detect DIF using non-IRT methods; while IRT-based methods for detecting DIF generally require larger sample sizes in order to estimate model parameters for both the reference and focal groups (Liu and Bradley, 2021; Ohiri et al., 2024).

In large sample sizes, regardless of group impact or the IRT model used, the Mantel–Haenszel (MH) method consistently outperformed IRT-based methods in terms of Type I error rates; furthermore, IRT-based DIF methods must account for correct model specification and group differences. The MH method showed greater stability than IRT methods, even under varying conditions of sample size and group impact (Diaz et al., 2021).

### 1.5 Aims of the present study

This study aimed to investigate if the admission test (PGAT) items have DIF based on gender for post-graduate students, and the type of DIF it may exhibit (uniform or non-uniform), and in which sub-tests (verbal, quantitative, and logical). In addition, it investigates DIF of PGAT items based on level of ability, to detect the items that may need to be adjusted or removed in order to have a fair test for both females and males. The following questions are addressed in this study:

1. Are there statistically significant differential performance (uniform or non-uniform) in the PGAT items across its scales (verbal, quantitative, logical and overall) based on gender, for post-graduate students? Would differences be reduced when DIF items are removed?
2. Does the differential performance of PGAT items across its scales (verbal, quantitative, logical and overall) differ at the high ability level, at which students are accepted, from the overall sample?

## 2 Method

### 2.1 Participants

The present study sample consisted of (*n* = 4,000) students randomly selected from a data set for the PGAT, administered by NCA in Saudi Arabia. The sample distributed equally between male and female groups according to gender variable: males (*n* = 2,000) and females (*n* = 2,000) that represented graduate students from universities across different regions of Saudi Arabia. The two groups were balanced to avoid any potential impact of sample imbalance on the accuracy of DIF estimation (Paek and Guo, 2011). Students who scored 60 or higher on the PGAT, totaling 676, were classified as a high-ability sample, as this score represents the official cutoff score adopted by universities for admission into postgraduate programs. The analysis was conducted on the total sample, and then on the high ability sample.

### 2.2 Measures

PGAT is a high-stake standardized test used for post graduate admission in Saudi Arabia, produced by the NCA (National Center for Assessment, 2015). It is similar to GRE, and is used to sort out the applicants for post-graduate studies. The test aims to identify students’ skills, which might predict success in post-graduate study and provide criteria by which post-graduate students can be selected. PGAT consists of (104) items divided into nine domains and three parts that measure number of abilities: The first part is verbal (linguistic), which includes (48) items that measure the ability to read with deep understanding and to distinguish the logical structure of linguistic expressions. The second part is quantitative (mathematical), which includes (24) items that measure the ability to solve problems based on basic mathematical concepts, and the abilities to infer and to conclude. While the third part is logical (inductive-spatial), which includes (32) items that measure the ability to perceive logical, spatial and non-spatial relationships, and the abilities to analyze, induct, and interpret the results. All questions are multiple-choice type. PGAT psychometric characteristics were evaluated by NCA and were acceptable (National Center for Assessment, 2015). We verified the evidence of validity and reliability of the test using several methods. The correlation coefficients between the scores on each sub-test of the test (verbal, quantitative, and logical) and the total scores were high (0.88, 0.78, and 0.82, respectively). All items showed acceptable and significant (*p* < 0.05) correlations with the sub-test scores they belong to, which provide evidence of test construct validity. The internal consistency of the overall test and sub-test scores were estimated by Cronbach’s alpha, and the values were (0.74, 0.64, 0.61, and 0.84) for females and (0.78, 0.67, 0.60, and 0.86) for males on verbal, quantitative, logical and the total scores respectively, which gives evidence of the reliability of the test.

### 2.3 Procedures

Data were collected from PGAT that was administered by NCA. All participants completed paper and pencil form of PGAT. The total time was 3 h, and administration procedures were standardized and controlled. NCA informed participants that their responses would be utilized in studies to evaluate test properties, and completion of the test is informed consent for their participation, and it guaranteed confidentiality of their personal information.

### 2.4 Analyses

Descriptive analysis was conducted to calculate test mean scores and standard deviations for males and females including total scores and scores of each sub-test, for the total sample as well as the high ability sample. Item analysis was then conducted to check item difficulty and item correlation with total and sub scores. The DIF analysis was conducted by Differential Item Performance Analysis System program (DIFAS 5.0; Penfield, 2013), which produces nonparametric DIF analyses using different procedures for dichotomously scored items. For DIF, test items were analyzed using the nonparametric Mantel–Haenszel (MH χ^2^) and Breslow-Day (BD χ^2^) statistics, which are based on chi-square distribution with 1 degree of freedom, with critical values of 3.84 (*α* = 0.05) and 6.63 (α = 0.01), to detect non-uniform DIF, in addition to Combined Decision Rule (CDR), where an item is flagged as showing potential DIF (“Yes”) if either the MH χ^2^ or BD χ^2^ statistic is significant at a Type I error rate of 0.025, and “No” means that neither of them shows s DIF (Penfield, 2013). We also used the Mantel–Haenszel Common Log-Odds Ratio (MH-LOR), to indicate whether an item favors either the reference group, which in the male group this study (positive values), or the target group, which in the female group this study (negative values). Values of the standardized log-odds ratio (LOR Z) greater than 2.0 or less than −2.0 may be considered evidence of the presence of DIF (Mantel and Haenszel, 1959). The higher the MH χ^2^ value, the higher the probability of test item to demonstrate DIF. For MH-LOR, it shows the magnitude of DIF, and whether DIF is uniform and affect one group consistently or non-uniform, depending on the value of the stratifying variable; and the higher value shows higher probability of DIF (Camilli and Shepard, 1994; Mantel and Haenszel, 1959). The Means and percentages of gender DIF items were summarized.

In addition, One Way Analysis of Variance (ANOVA) was conducted to compare performances on total and sub-scores between males and females, in addition to effect size, for both overall sample and high ability sample. ANOVA analysis was conducted before and after deleting items that showed DIF. ANOVA was used to compare the means of the two groups (males and females) controlling for type I error, as it is equivalent to a t-test, which is a special case of ANOVA (Ross and Willson, 2017). For effect size, Eta-Squared (η^2^) was applied, and it was considered large if (≥ 0.14), medium if (≤0.13–0.06), and small if (>0.06) (Cohen, 1988).

## 3 Results

Several nonparametric statistics (MH χ^2^, MH-LOR, BD χ^2^, and CDR) were used in this study to investigate DIF in PGAT items, based on gender; in addition to item difficulties (*p*), and corrected item-total score correlations (ritc), for the overall sample. Then the same analyses were repeated for the selected high ability sample; to see if they share the same results, as in Table 1. Besides, Table 2 shows numbers and percentages of these gender DIF statistics.

**Table 1:** Gender DIF statistics in PGAT items using (MH *χ*^2^, MH-LOR, BD *χ*^2^, and CDR methods), item difficulties (*p*), and corrected item-total correlations (*r*_itc_) for the overall sample and the high ability sample.

Item	Overall sample						High ability sample					
	MH *χ*^2^	MH LOR	BD *χ*^2^	CDR	*p*	*r*_itc_	MH *χ*^2^	MH LOR	BD *χ*^2^	CDR	*p*	*r*_itc_
Ver1	10.78	−0.23	1.65	Yes	0.45	0.32	1.18	−0.20	0.78	No	0.73	0.05
Ver2	23.67	0.36	7.84	Yes	0.36	0.36	7.23	0.48	5.34	Yes	0.69	0.13
Ver3	104.44	−0.73	5.49	Yes	0.67	0.22	20.74	−1.02	1.21	Yes	0.83	−0.01
Ver4	39.84	0.49	0.88	Yes	0.23	0.10	7.63	0.49	3.40	Yes	0.31	0.02
Ver5	1.83	−0.09	3.31	No	0.46	0.25	3.38	−0.32	0.02	No	0.66	0.02
Ver6	0.15	−0.03	0.01	No	0.32	0.14	1.15	−0.18	2.30	No	0.43	−0.19
Ver7	47.66	−0.46	1.90	Yes	0.51	0.17	8.08	−0.48	0.68	Yes	0.63	−0.05
Ver8	8.29	−0.20	0.09	Yes	0.39	0.21	0.08	0.06	0.98	No	0.59	0.07
Ver9	0.55	0.05	3.51	No	0.42	0.08	3.25	0.30	0.00	No	0.51	0.07
Ver10	6.26	−0.18	1.13	No	0.40	0.39	3.42	−0.35	0.44	No	0.71	0.06
Ver11	4.54	−0.16	0.05	No	0.29	0.25	1.49	−0.20	0.10	No	0.52	0.04
Ver12	1.31	0.08	3.44	No	0.33	0.10	2.06	0.25	0.05	No	0.44	0.03
Ver13	3.35	−0.15	1.81	No	0.20	0.11	1.04	−0.20	0.09	No	0.30	0.08
Ver14	1.71	0.11	0.00	No	0.20	0.13	0.29	0.11	0.01	No	0.34	0.12
Ver15	3.46	0.13	1.70	No	0.57	0.19	0.07	0.06	0.01	No	0.72	−0.02
Ver16	2.42	0.11	0.82	No	0.43	0.24	4.20	0.35	4.61	No	0.64	0.08
Ver17	4.57	−0.15	0.86	No	0.60	0.25	3.56	−0.38	0.26	No	0.79	0.09
Ver18	6.20	−0.18	1.66	No	0.59	0.35	1.46	−0.29	0.32	No	0.85	0.06
Ver19	0.40	0.05	0.31	No	0.38	0.25	0.00	0.00	2.13	No	0.61	0.05
Ver20	9.79	−0.28	0.41	Yes	0.83	0.22	0.91	−0.42	0.00	No	0.95	0.02
Ver21	5.73	−0.17	1.55	No	0.69	0.23	0.95	−0.26	0.78	No	0.87	0.02
Ver22	9.62	−0.23	0.45	Yes	0.70	0.27	1.63	−0.31	0.63	No	0.86	0.08
Ver23	9.79	−0.23	0.30	Yes	0.69	0.23	0.19	−0.12	0.81	No	0.84	0.05
Ver24	1.04	−0.11	0.78	No	0.87	0.24	0.10	−0.22	2.68	No	0.96	0.04
Ver25	0.39	−0.04	0.02	No	0.47	0.27	1.46	−0.24	1.27	No	0.75	0.09
Ver26	14.13	0.26	3.52	Yes	0.52	0.30	3.85	0.40	1.99	No	0.78	0.08
Ver27	0.06	0.02	8.99	Yes	0.53	0.31	0.41	0.14	1.47	No	0.80	0.08
Ver28	6.91	−0.19	6.04	Yes	0.38	0.32	0.85	−0.17	0.54	No	0.66	0.16
Ver29	1.97	−0.10	0.06	No	0.66	0.32	0.37	0.21	0.12	No	0.91	0.09
Ver30	89.03	−0.67	0.03	Yes	0.65	0.26	0.65	−0.21	1.56	No	0.86	0.09
Ver31	2.70	−0.12	0.02	No	0.62	0.33	0.02	−0.06	0.15	No	0.86	0.07
Ver32	4.30	−0.15	0.39	No	0.67	0.31	0.17	−0.13	0.85	No	0.87	0.02
Ver33	46.01	−0.52	0.32	Yes	0.76	0.08	4.86	−0.50	0.50	No	0.84	0.01
Ver34	21.19	−0.51	0.78	Yes	0.90	0.16	2.99	−0.81	1.72	No	0.96	0.05
Ver35	0.00	0.01	0.10	No	0.19	−0.09	0.12	−0.10	1.61	No	0.14	−0.09
Ver36	4.39	0.15	0.06	No	0.45	0.36	0.29	0.12	5.17	No	0.77	0.11
Ver37	2.34	0.11	1.07	No	0.36	0.27	4.70	0.36	4.62	No	0.61	0.04
Ver38	0.02	−0.02	7.49	Yes	0.19	0.20	0.45	−0.12	0.50	No	0.36	0.09
Ver39	15.08	−0.51	0.55	Yes	0.92	0.25	0.74	0.97	1.74	No	0.99	0.01
Ver40	81.46	−0.83	0.08	Yes	0.83	0.24	2.29	−0.64	2.28	No	0.95	0.03
Ver41	10.43	−0.23	1.85	Yes	0.67	0.17	3.10	−0.35	2.24	No	0.78	−0.01
Ver42	61.58	−0.65	0.05	Yes	0.76	0.34	1.42	−0.46	0.52	No	0.94	0.04
Ver43	11.81	−0.22	0.15	Yes	0.46	0.06	0.67	−0.14	2.94	No	0.51	−0.10
Ver44	12.90	0.31	0.41	Yes	0.18	−0.17	0.74	−0.25	0.01	No	0.11	−0.11
Ver45	8.97	−0.20	1.34	Yes	0.50	0.21	3.55	−0.32	7.84	Yes	0.64	0.05
Ver46	130.74	0.79	0.00	Yes	0.54	0.28	12.26	0.70	0.59	Yes	0.77	0.11
Ver47	4.84	−0.15	1.19	No	0.61	0.09	1.65	−0.24	0.19	No	0.70	0.09
Ver48	0.50	−0.05	0.15	No	0.46	0.21	0.58	−0.14	0.31	No	0.63	0.10
Qun1	0.47	0.05	6.73	Yes	0.64	0.28	0.09	−0.09	3.56	No	0.86	0.08
Qun2	7.89	0.22	0.02	Yes	0.76	0.25	0.31	0.18	0.43	No	0.91	0.08
Qun3	21.14	0.33	5.96	Yes	0.60	0.36	0.01	0.00	0.33	No	0.87	0.04
Qun4	3.84	−0.14	4.87	No	0.38	0.25	0.00	0.01	0.38	No	0.64	0.18
Qun5	1.29	−0.09	6.15	No	0.26	0.02	1.17	0.20	3.94	No	0.34	0.06
Qun6	15.53	0.28	9.61	Yes	0.35	0.31	13.69	0.64	6.15	Yes	0.67	0.10
Qun7	0.04	0.02	2.35	No	0.38	0.08	0.86	0.16	3.22	No	0.49	0.06
Qun8	0.02	0.01	1.41	No	0.40	0.26	0.03	0.05	0.01	No	0.66	0.11
Qun9	12.35	0.25	9.47	Yes	0.29	0.11	13.86	0.62	2.04	Yes	0.40	0.10
Qun10	3.32	0.13	0.84	No	0.34	0.32	0.23	0.09	4.16	No	0.66	0.11
Qun11	9.53	0.23	1.24	Yes	0.39	0.42	0.04	0.06	4.63	No	0.81	0.17
Qun12	2.05	0.10	0.49	No	0.37	0.23	0.01	−0.03	1.23	No	0.63	0.21
Qun13	21.26	0.34	10.72	Yes	0.28	0.23	17.94	0.70	0.13	Yes	0.51	0.15
Qun14	1.02	−0.08	0.15	No	0.26	0.12	0.01	0.03	0.03	No	0.40	0.13
Qun15	70.54	0.58	10.90	Yes	0.53	0.29	16.63	0.82	1.05	Yes	0.79	0.04
Qun16	39.21	0.44	10.62	Yes	0.43	0.35	15.65	0.77	4.16	Yes	0.78	0.12
Qun17	0.00	0.01	0.24	No	0.70	0.34	0.23	0.17	0.08	No	0.91	0.04
Qun18	1.35	0.10	0.00	No	0.77	0.30	0.01	−0.08	0.01	No	0.94	0.01
Qun19	21.31	−0.38	0.02	Yes	0.21	0.12	6.22	−0.42	2.85	No	0.36	0.11
Qun20	0.23	−0.05	2.09	No	0.13	−0.06	1.69	−0.35	0.15	No	0.12	−0.10
Qun21	0.01	0.01	6.65	Yes	0.10	0.08	4.26	−0.46	0.98	No	0.16	0.05
Qun22	8.30	0.23	0.16	Yes	0.24	0.25	0.81	0.16	0.41	No	0.52	0.16
Qun23	0.33	−0.05	16.03	Yes	0.22	0.08	11.65	−0.60	1.59	Yes	0.30	−0.08
Qun24	9.50	0.24	5.99	Yes	0.24	0.20	6.16	0.41	4.49	No	0.43	0.11
Logic1	0.51	0.05	0.51	No	0.32	0.12	0.06	0.05	0.28	No	0.43	−0.03
Logic2	65.95	0.59	1.35	Yes	0.29	0.05	13.77	0.63	0.00	Yes	0.36	−0.01
Logic3	0.40	0.05	0.87	No	0.35	0.24	0.05	−0.05	0.01	No	0.58	0.09
Logic4	14.33	0.38	0.22	Yes	0.87	0.16	2.46	0.55	0.44	No	0.93	0.00
Logic5	32.14	0.37	0.30	Yes	0.42	−0.10	4.44	0.36	2.22	No	0.37	−0.11
Logic6	338.68	1.25	5.05	Yes	0.40	0.05	44.90	1.11	1.88	Yes	0.46	0.03
Logic7	6.33	0.17	2.55	No	0.62	0.22	4.80	0.46	2.57	No	0.81	0.01
Logic8	21.06	0.38	0.01	Yes	0.19	−0.01	0.30	0.13	0.14	No	0.18	−0.08
Logic9	29.93	−0.36	0.18	Yes	0.42	0.11	4.16	−0.33	7.90	Yes	0.52	0.00
Logic10	87.97	0.63	0.33	Yes	0.58	0.14	19.16	0.78	0.16	Yes	0.70	−0.08
Logic11	0.00	0.01	1.46	No	0.43	0.21	0.62	−0.14	0.13	No	0.61	0.05
Logic12	23.00	0.32	6.08	Yes	0.39	0.16	5.53	0.39	1.05	No	0.58	0.02
Logic13	1.90	−0.11	2.08	No	0.23	0.20	0.01	0.03	2.01	No	0.44	0.05
Logic14	2.98	−0.12	0.02	No	0.63	0.10	0.26	−0.10	0.00	No	0.71	−0.04
Logic15	0.05	−0.02	4.06	No	0.20	0.23	2.75	−0.27	3.34	No	0.42	0.05
Logic16	0.95	−0.07	7.80	Yes	0.47	0.38	3.23	−0.39	1.59	No	0.81	0.11
Logic17	12.51	−0.25	3.81	Yes	0.66	0.14	5.10	−0.46	0.95	No	0.78	−0.09
Logic18	5.87	0.17	0.38	No	0.60	0.32	1.50	0.32	0.01	No	0.88	0.04
Logic19	10.28	−0.22	0.06	Yes	0.60	0.24	1.66	−0.26	2.00	No	0.80	0.03
Logic20	5.87	0.22	0.07	No	0.16	0.02	0.13	0.09	1.29	No	0.20	0.02
Logic21	7.07	−0.18	5.09	Yes	0.44	0.14	3.22	−0.30	6.19	No	0.61	0.10
Logic22	3.22	−0.13	5.71	No	0.61	0.33	7.07	−0.68	3.39	Yes	0.87	0.10
Logic23	0.62	−0.07	1.84	No	0.79	0.26	0.46	−0.29	0.01	No	0.94	0.04
Logic24	13.28	−0.24	3.87	Yes	0.59	0.15	4.92	−0.41	0.00	No	0.72	−0.06
Logic25	22.20	0.33	2.94	Yes	0.37	0.28	0.18	−0.08	1.12	No	0.65	0.17
Logic26	5.12	−0.17	2.70	No	0.51	0.43	0.89	−0.25	2.38	No	0.87	0.15
Logic27	2.62	0.12	0.01	No	0.44	0.37	1.52	0.25	0.74	No	0.77	0.09
Logic28	26.96	−0.35	0.02	Yes	0.53	0.20	2.70	−0.30	0.18	No	0.70	0.01
Logic29	5.32	0.16	0.08	No	0.32	0.15	0.48	0.12	0.04	No	0.47	0.08
Logic30	12.53	−0.24	6.90	Yes	0.45	0.21	19.65	−0.77	7.96	Yes	0.67	0.09
Logic31	11.04	−0.22	10.02	Yes	0.50	0.24	8.74	−0.53	8.49	Yes	0.72	0.08
Logic32	30.41	−0.37	4.00	Yes	0.56	0.20	8.32	−0.53	7.43	Yes	0.74	0.06

CDR: Yes, DIF is present; No, No DIF is present; Positive values for MH-LOR indicate DIF is in favor of the reference group (males), and negative values indicate DIF is in favor of the focal group (females). Ver, Verbal ability; Quan, Quantitative ability; Logic, Logical ability.

Shading was used to highlight the items showing DIF and to facilitate their tracking.

**Table 2:** Numbers and percentages of gender DIF statistics in PGAT items using (MH *χ*^2^, MH-LOR, BD *χ*^2^, and CDR methods), item difficulties (*p*), and corrected item-total correlations (*r*_itc_) for the overall sample and the high ability sample.

Sample	PGAT domain	Number and percentage of DIF items (MH *χ*^2^)	Number and percentage of DIF items favoring females (MH *χ*^2^)	Number and percentage of DIF items favoring males (MH *χ*^2^)	Non-uniform DIF (BD *χ*^2^)	*p* mean (Min, Max)	*r*_itc_ mean (Min, Max)
Overall sample	The verbal sub-test (linguistic)	24/48 (50%)	17 (71%)	5 (20%)	2 (9%)	0.52 (0.18, 0.92)	0.23 (−0.17, 0.39)
	The quantitative sub-test (mathematical)	14/24 (58%)	1 (7%)	10 (71%)	3 (22%)	0.39 (0.1, 0.77)	0.23 (−0.06, 0.42)
	The logical sub-test (inductive-spatial)	18/32 (56%)	9 (50%)	8 (44%)	1 (6%)	0.47 (0.16, 0.87)	0.19 (−0.1, 0.43)
	Overall PGAT	56/104 (54%)	27 (48%)	23 (41%)	6 (11%)	0.47 (0.1, 0.92)	0.21 (−0.17, 0.43)
High ability sample	The verbal sub-test (linguistic)	6/48 (13%)	2 (33%)	3 (50%)	1 (17%)	0.69 (0.11, 0.99)	0.04 (−0.19, 0.16)
	The quantitative sub-test (mathematical)	6/24 (25%)	1 (17%)	5 (83%)	–	0.59 (0.12, 0.94)	0.09 (−0.1, 0.21)
	The logical sub-test (inductive-spatial)	8/32 (25%)	5 (62%)	3 (38%)	–	0.63 (0.18, 0.94)	0.03 (−0.11, 0.17)
	Overall PGAT	20/104 (19%)	8 (40%)	11 (55%)	1 (5%)	0.65 (0.11, 0.99)	0.05 (−0.19, 0.21)

For the overall sample, Tables 1, 2 show that based on gender, there were 56 out of 104 (54%) items of PGAT that had DIF according to (CDR) method (based on one or more of DIF indicators: MH χ^2^, and BD χ^2^); 24 out of 48 (50%); 14 out of 24 (58%); and 18 out of 32 (56%) on verbal, quantitative and logical abilities, respectively. There were 27 (48%) items showed DIF in favor of females, and 23 (41%) items showed DIF in favor of males; and 6 (11%) of the items showed non-uniform DIF.

In verbal ability, 5 (20%) of DIF items were in favor of males (ver2, ver4, ver27, ver44, and ver46), and 17 (71%) were in favor of females (ver1, ver3, ver7, ver8, ver20, ver22, ver23, ver28, ver30, ver33, ver34, ver39, ver40, ver41, ver42, ver43, and ver45); and two of the items (9%)(ver27, ver38) showed non-uniform DIF. While, in quantitative ability, 10 (71%) of the items with DIF were in favor of males (qun2, qun3, qun6, qun9, qun11, qun13, qun15, qun16, qun22, and qun24), and one item (7%) was in favor of females (qun19), and three of the items (22%) (qun1, qun21, qun23) showed non-uniform DIF. Furthermore, in the logical ability, 8 (44%) of the items with DIF were in favor of males (logic2, logic4, logic5, logic6, logic8, logic10, logic12 and logic25), and 9 (50%) were in favor of females (logic9, logic17, logic19, logic21, logic24, logic28, logic30, logic31, and logic32), and one of the items (6%)(Logic16) showed non-uniform DIF.

For the high ability sample, Tables 1, 2 show that there were 20 out of 104 (19%) items of PGAT that had DIF according to (CDR) method (based on one or more of DIF indicators: MH χ^2^, and BD χ^2^); 6 out of 48 (13%); 6 out of 24 (25%); and 8 out of 32 (25%) on verbal, quantitative and logical abilities, respectively. These results represent a high decrease in the number of items that showed DIF from (56) for overall sample to (20) for high ability sample (54 to 19%). There were 8 (40%) of DIF items in favor of females, and 11 (55%) in favor of males, and one of the items was with non-uniform DIF.

In the verbal ability, 3 (50%) of DIF items were in favor of males (ver2, ver4, and ver46), and 2 (33%) in favor of females (ver3 and ver7), and one item (17%)(ver45) showed non-uniform DIF. On the other side, in the quantitative ability, 5 (83%) of DIF items were in favor of males (qun6, qun9, qun13, qun15, and qun16), and one (17%) was in favor of females (qun23), while no item showed non-uniform DIF. Moreover, in the logical ability, 3 (38%) of DIF items were in favor of males (logic2, logic6, and logic10), and 5 (62%) were in favor of females (logic9, logic22, logic30, logic31, and logic32), while no item showed non-uniform DIF.

In addition, item correlations with the total scores were higher for overall sample compared to high ability sample. In contrast, *p* values for DIF were higher for high ability sample compared to overall sample; and DIF appeared on difficult as well as easy items, ranging from (0.11 to 0.96).

To examine the differences between males and females in the performance on PGAT total and sub-tests, one way ANOVA was conducted on the overall sample, then it was repeated after the elimination of DIF items using (CDR) method; to see if differences could be reduced when DIF items are removed. The same analyses was conducted for the high ability sample to see if the same or different patterns of differences would appear compared to overall sample. Table 2 shows means, standard deviations, ANOVA results, and the effect size on PGAT total and sub-tests, for the overall sample as well as the high ability sample, before and after eliminating DIF items.

For the overall sample, the results in Table 2 show that females scored higher than males on verbal ability sub-test (females: M = 25.48, SD = 6.07; males: M = 24.23, SD = 6.58), and on the overall test score (females: M = 49.31, SD = 11.46; males: M = 48.74, SD = 12.18). On the other side, males scored higher than females on quantitative and logical abilities sub-tests (males: M = 9.47, SD = 3.71, and M = 15.04, SD = 4.13; females: M = 8.99, SD = 3.48, and M = 14.84, SD = 4.18), respectively.

ANOVA results for the overall sample showed statistically significant differences (*p* < 0.01) between the two groups in verbal ability, in favor of females; and in quantitative ability, in favor of males; while there were no statistically significant differences between the two groups (*p* > 0.05) on the logical ability sub-test and overall test score. These results were before deleting DIF items. After deleting items that showed DIF according to (CDR) for the overall sample, the results revealed that females still scored higher than males on verbal ability (females: M = 11.43, SD = 3.53; males: M = 11.08, SD = 3.69), quantitative ability (females: M = 3.98, SD = 1.68; males: M = 3.96, SD = 1.79), and on the overall test score (females: M = 21.62, SD = 6.02; males: M = 21.25, SD = 6.31), respectively. On the other side, males scored equal to females on logical ability sub-test (females: M = 6.21, SD = 2.34; males: M = 6.21, SD = 2.38). All differences between males and females were not statistically significant (*p* > 0.05), except on verbal ability which was in favor of females. All differences were with very small effect sizes (d < 0.06).

For the high ability sample, the results in Table 2 shows that females scored higher than males on verbal ability sub-test (females: M = 33.12, SD = 3.29; males: M = 32.82, SD = 4.11). On the other side, males scored higher than females on quantitative and logical abilities sub-tests as well as the overall test score (for males: M = 14.54, SD = 2.99; M = 20.32, SD = 2.72; and M = 67.67, SD = 5.96; and for females: M = 13.73, SD = 2.95; M = 20.29, SD = 2.77; and M = 67.15, SD = 5.01), respectively.

ANOVA results for the high ability sample showed statistically significant differences (*p* < 0.01) between the two groups in quantitative ability, in favor of males; while there were no statistically significant differences (*p* > 0.05) on verbal and logical abilities as well as the total test score. All differences were with very small effect sizes (d < 0.06), with somewhat higher effect size on the quantitative part.

After deleting the items that showed DIF according to (CDR) for the high ability sample, the results revealed that females still scored higher than males on verbal ability sub-test (females: M = 29.26, SD = 2.99; males: M = 28.94, SD = 3.69), logical ability part (females: M = 15.28, SD = 2.23; males: M = 15.25, SD = 2.21), and on the overall test score (females: M = 55.15, SD = 4.11; males: M = 54.99, SD = 5.21), respectively. On the other side, males scored higher than females on quantitative ability sub-test (males: M = 10.80, SD = 2.56; females: M = 10.61, SD = 2.48). All differences between males and females were not statistically significant in verbal, quantitative, logical abilities and PGAT overall scores (*p* > 0.05) and were with very small effect sizes (d < 0.06) (see Table 3).

**Table 3:** Means (M), standard deviations (SD), and one way ANOVA results for the differences between males (*n* = 2,000) and females (*n* = 2,000) on PGAT scores, before and after the elimination of DIF items for overall and high ability samples using (CDR) method.

Sample	PGAT domain	Number of DIF items	Females		Males		ANOVA		
			M	SD	M	SD	*F*	*p*	*d*
Overall sample	(A) PGAT before eliminating DIF items								
	The verbal sub-test (linguistic)	48	25.48	6.07	24.23	6.58	39.21	0.001**	0.01
	The quantitative sub-test (mathematical)	24	8.99	3.48	9.47	3.71	17.85	0.001**	0.004
	The logical sub-test (inductive-spatial)	32	14.84	4.18	15.04	4.13	2.41	0.121	0.004
	Overall PGAT	104	49.31	11.46	48.74	12.18	2.28	0.127	0.001
	(B) PGAT after eliminating DIF items (CDR)								
	The verbal sub-test (linguistic)	24/48	11.43	3.53	11.08	3.69	9.28	0.002**	0.002
	The quantitative sub-test (mathematical)	14/24	3.98	1.68	3.96	1.79	0.14	0.709	0.000
	The logical sub-test (inductive-spatial)	18/32	6.21	2.34	6.21	2.38	0.01	0.909	0.000
	Overall PGAT	56/104	21.62	6.02	21.25	6.31	3.73	0.053	0.001
High ability sample	(C) PGAT before eliminating DIF items								
	The verbal sub-test (linguistic)	48	33.12	3.29	32.82	4.11	1.07	0.302	0.002
	The quantitative sub-test (mathematical)	24	13.73	2.95	14.54	2.99	12.28	0.001**	0.018
	The logical sub-test (inductive-spatial)	32	20.29	2.77	20.32	2.72	0.010	0.918	0.000
	Overall PGAT	104	67.15	5.01	67.67	5.96	1.54	0.216	0.002
	(D) PGAT after eliminating DIF items (CDR)								
	The verbal sub-test (linguistic)	6/48	29.26	2.99	28.94	3.69	1.52	0.218	0.002
	The quantitative sub-test (mathematical)	6/24	10.61	2.48	10.80	2.56	1.03	0.311	0.002
	The logical sub-test (inductive-spatial)	8/32	15.28	2.23	15.25	2.21	0.04	0.851	0.000
	Overall PGAT	20/104	55.15	4.11	54.99	5.21	0.18	0.668	0.000

*F* = ANOVA test value; **p < 0.01; *d* = effect size.

## 4 Discussion

In the present study, we investigated gender differences on PGAT (verbal, quantitative, logical, and overall scores) for post-graduate students, whether they are real differences or due to DIF, and if gender gap could be reduced when DIF items are removed, or when high ability sample is used, with scores higher that cut score for acceptance in post-graduate programs.

Regarding PGAT total and sub-scale scores; for the overall sample, females outperformed males on overall score and on verbal ability, while males outperformed females on the quantitative ability, significantly. These results are partially in accordance with the results from previous research (Ajmi et al., 2023; Al-Bursan, 2013; Al-Bustanji, 2004; Almaskari et al., 2021; Hyde and Linn, 1988; Hyde and Mertz, 2009; Kalaycioglu and Berberoglu, 2011; Shamsaldeen et al., 2024; Setiawan et al., 2024; Yörü and Atar, 2019; Wedman, 2018) that reported significant differences based on gender on cognitive tests, where women outperformed men on verbal abilities, and men outperformed women on quantitative and logical abilities, with considerable magnitude. However, this general belief does not apply at all ability levels as it was showed in this study. Furthermore, this study was conducted on post-graduate admission test and considered methodological aspects regarding gender real differences and the ones due to DIF.

When DIF items were removed for the overall sample in this study, gender differences on PGAT scores were greatly reduced and were not statistically significant, except for verbal ability where differences continued to favor females and were statistically significant. In addition, females continued to outperform males on overall test scores. Unexpectedly, females also outperformed males slightly in quantitative scores, and were equal to them on logical scores, after removing DIF items.

For the high ability sample, females outperformed males only on verbal ability, while males outperformed females on quantitative and logical abilities as well as overall score. However, all these differences were not statistically significant, except for quantitative ability. The magnitudes of the differences were in general low. When DIF items were removed, females continued to outperform males on verbal ability and overall score, and males continued to outperform females on quantitative ability. The results were reversed on logical ability for high ability sample, where females outperformed males slightly, after removing DIF items. All differences were not statistically significant including quantitative ability. This indicated that gender differences in performance on PGAT differ based on ability level. In addition, it appeared that the differences based on gender in verbal ability are real, in favor of females; while differences in quantitative ability are not real, because they were not significant after removing DIF items; and differences in logical ability are not real either, because they were not significant before nor after removing DIF items.

Regarding PGAT items, for the overall sample, more than half of the items (54%) showed DIF, ranging from (50 to 58%) within sub-tests, which could be considered high percentages; and most of them (48%) were in favor of females. In comparison, for the high ability sample, the percentage of total DIF items decreased to (19%), ranging from (13 to 25%) within sub-tests, which could be considered low percentages; and most of them (55%) were in favor of males. The highest decrease was on verbal sub-test (from 50 to 13%). Moreover, PGAT items showed some non-uniform DIF across gender and ability, for all PGAT sub-tests (verbal, logical and quantitative abilities). This result agrees to some level with the studies conducted on undergraduate admission tests in Saudi Arabia (e.g., Sideridis and Tsaousis, 2013a, 2013b; Tsaousis et al., 2020, 2023), that reported uniform and non-uniform DIF for both males and females on these tests.

Gender DIF items results of PGAT interacted with ability level. When analysis was conducted on high ability sample, DIF results changed; where percentage of DIF items favoring females decreased (from 48 to 40%), particularly on verbal ability (from 71 to 50%); while the percentage of DIF items favoring males increased (from 41 to 55%), particularly on verbal (from 21 to 50%) and quantitative ability (from 71 to 83%). This result supports in part other studies (Al-Bursan, 2013; Shanmugam, 2020) that reported that DIF direction and percentage may differ in cognitive tests based on ability level. Additionally, this result is partially consistent with Al-Bursan’s (2013) study which indicated that the percentage of DIF items based on gender increased as student ability decreased, where there was less DIF items for high ability levels mostly favoring females vs. more DIF for low ability levels mostly favoring males. However this study results did not support the conclusion that gender differences in quantitative ability were most at the very high levels of ability.

This study also coincides with other studies (such as: Abedalaziz, 2010; Al-Bustanji, 2004; Chiu, 2008; Hambleton and Rogers, 1989; Kalaycioglu and Berberoglu, 2011; Kelecioglu et al., 2014; Pedrajita and Talisayon, 2009; Penfield, 2003; Salubayba, 2013) that used combined MH with other methods (such as BD) to detect the uniform and non-uniform DIF effectively.

For verbal ability, the study results showed that females outperformed males across ability levels, even after removing DIF items. However, the percentage of verbal ability DIF items became almost equal between males and females at high ability level. This result does not support other studies (Abedalaziz, 2010; Wedman, 2018), that indicated that most verbal ability items that showed DIF were in favor of females. For quantitative ability, the study showed that gender differences were reversed in favor of females, once DIF items were removed for the overall sample; while they remain favoring males for the high ability sample. The majority of items that showed DIF were in favor of males for overall sample as well as high ability sample. As far as logical ability, the study showed that DIF items were in favor of females for overall sample as well as the high ability sample. These results are in accordance with other studies (Abedalaziz, 2010; Al-Bursan, 2013; Al-Bustanji, 2004; Kalaycioglu and Berberoglu, 2011; Yörü and Atar, 2019), which revealed that most of the items that showed DIF on mathematical ability were favoring male group. However, the results partially contradict with Shanmugam’s (2020) study results that suggested that simple mathematical items favored females while higher-order thinking items favored males.

This study results might also be explained by the sample that we investigated, which included post graduate students with heterogenous performance, and that some female as well as male students applying for graduate studies may have had very high abilities regardless of gender, which may have affected the results.

These results imply that different DIF analysis should be conducted for different groups based on gender and ability levels, with more focus on intended groups. For example, PGAT intended group is students with high score (60 and above) who will be accepted at post-graduate programs. The focus of DIF analysis should consider this group, more than all students with different abilities. Including other groups with lower scores may give mix DIF results, causing elimination of items that have no DIF for intended groups, and thus affecting the test accuracy.

In general, the results of this study indicated that gender differences on PGAT could largely be attributed to DIF, and that gender gap was reduced once DIF items were removed, except for gender differences in verbal ability that remained after removing DIF items. However, gender gab seems to be overestimated due to the large number of items with DIF, which could be attributed to applying the MH method to a large sample size, as Alomari et al. (2023) indicated. Other factors could also have affected these results of DIF detection, including: test length, the magnitude of DIF, and the percentage of items exhibiting DIF. Further studies need to be conducted with more manipulation on these factors and type I error rates. Item content could be one of the reasons, however the researchers do not have access to it. De Ayala (2009) indicated that while it is difficult to ascertain the reasons for DIF, it is up to the specialists to decide whether to treat DIF items or replace them.

Finally, there are two limitations in this study. Since the PGAT items remain confidential and were not provided to us, we could not perform a substantive review of the item content to further explore the reasons that caused DIF. Content experts should review the DIF items in the test to identify possible sources of DIF and provide “explainable sources of bias” for removing or revising any DIF items. Multilevel methods of DIF could be used to account for different conditions including multilevel MH, when latent means are not equal for the groups (Valdivia et al., 2024).

## 5 Conclusion

The study showed that there were gender differences on PGAT and they could largely be attributed to DIF. Gender gap was reduced when DIF items were removed. Percentage of DIF items decreased across sub-tests, when ability increased, particularly for females on verbal sub-test. The study results also showed that gender DIF interacted with ability level, and differed for high ability group than the overall sample, particularly on verbal sub-test. Consequently, for ability tests it is necessary to conduct stratified DIF analysis based on ability area, gender, ability level and their interaction. In addition, it is recommended to report DIF for targeted ability groups, with its size and direction.

In light of the study findings, it is recommended for admission tests to consider the cut scores in conducting DIF analysis in order to fit excluded and included items with the target group. This study should be replicated on Post-Graduate Aptitude Tests, such as GRE, to examine whether the same results could be obtained in other countries.

Some limitations of this study include the use of a single DIF detection method, the application of fixed and balanced sample sizes, and the inability to access item content due to test security restrictions. It is the responsibility of the test developers and experts to consider the results of the study, and to investigate the causes behind the observed differential performance on certain items of the PGAT, in accordance with the policy of maintaining test content confidentiality.

Furthermore, this study focused exclusively on the Mantel–Haenszel (MH) procedure in investigating DIF of PGAT items, based on ability levels. The performance of other DIF methods, particularly those grounded in IRT, in detecting DIF based on ability levels is not included in this study, and could be considered in future research. Additionally, the effectiveness of DIF detection methods based on ability levels could be further examined in with samples that involve varying levels of ability and highly unbalanced sample sizes.

Consequently, the findings of this study may be replicated using advanced DIF approaches that control for ability levels using IRT framework indicators (Lim et al., 2021), as well as Differential Bundle Functioning (DBF) methods, wherein items are grouped into bundles for DIF analysis (Li and Becker, 2021).

## Data Availability

The raw data supporting the conclusions of this article will be made available by the authors, without undue reservation.
